# Comprehensive Analysis of LINC01615 in Head and Neck Squamous Cell Carcinoma: A Hub Biomarker Identified by Machine Learning and Experimental Validation

**DOI:** 10.1155/2022/5039962

**Published:** 2022-06-27

**Authors:** Xiaoyan Yin, Jingmiao Wang, Yanrui Bian, Qiaojing Jia, Ziyi Shen, Haizhong Zhang

**Affiliations:** Department of Otolaryngology, Head and Neck Surgery, The Second Hospital Of Hebei Medical University, Shijiazhuang, China

## Abstract

**Background:**

Head and neck squamous cell carcinoma (HNSCC) is one of the most common cancers, but in clinical practice, the lack of precise biomarkers often results in an advanced diagnosis. Hence, it is crucial to explore novel biomarkers to improve the clinical outcome of HNSCC patients.

**Methods:**

We downloaded RNA-seq data consisting of 502 HNSCC tissues and 44 normal tissues from the TCGA database, and lncRNA genomic sequence information was downloaded from the GENECODE database for annotating lncRNA expression profiles. We used Cox regression analysis to screen prognostic lncRNAs, the threshold as HR >1 and *p* value <0.05. Subsequently, three survival outcomes (overall survival, progress-free interval, and disease-specific survival)-related lncRNAs overlapped to get the common lncRNAs. The hub biomarker was identified using LASSO and random forest models. Subsequently, we used a variety of statistical methods to validate the prognostic ability of the hub marker. In addition, Spearman correlation analysis between the hub marker expression and genomic heterogeneity was conducted, such as instability (MSI), homologous recombination deficiency (HRD), and tumor mutational burden (TMB). Finally, we used enrichment analysis, ssGSEA, and ESTIMATE algorithms to explore the changes in the underlying immune-related pathway and function. Finally, the MTT assay and transwell assay were performed to determine the effect of LINC01615 silencing on tumor cell proliferation, invasion, and migration.

**Results:**

Cox regression analysis revealed 133 lncRNAs with multiple prognostic significance. The machine learning algorithm screened out the hub lncRNA with the highest importance in the RF model: LINC01615. Clinical correlation analysis revealed that the LINC01615 increased with increasing the T stage, N stage, pathology grade, and clinical stage. LINC01615 could be used as a predictor of HNSCC prognosis validating by a variety of statistical methods. Subsequently, when clinical indicators were combined with the LINC01615 expression, the visualization model (nomogram) was more applicable to clinical practice. Finally, immune algorithms indicated that LINC01615 may be involved in the regulation of lymphocyte recruitment and immunological infiltration in HNSCC, and the LINC01615 expression represented genomic heterogeneity in pan-cancer. Functionally, silencing of LINC01615 suppresses cell proliferation, invasion, and migration in HEP-2 and TU212 cells.

**Conclusion:**

LINC01615 may play an important role in the prostromal cell enrichment and immunosuppressive state and serve as a prognostic biomarker in HNSCC.

## 1. Introduction

Head and neck squamous cell carcinoma (HNSCC) can occur in different tissues and organs (oral cavity, oropharynx, and larynx regions) [1], and the illness ranks sixth in global incidence according to the recent cancer statistics report [2]. Although several clinical trials have shown that combining different treatment modalities, such as surgery, radiation, and chemotherapy, can significantly reduce the mortality and improve the life quality of HNSCC patients [3], the situation remains dire. The 5-year overall survival rate of HNSCC is about 50% [4], but in clinical practice, the lack of precise biomarkers often results in an advanced diagnosis. Hence, it is crucial to explore novel and effective treatment strategies to improve the clinical outcome of HNSCC.

Based on the protein coding ability, RNA can be classified into the coding RNA and the noncoding RNA (ncRNA). Long noncoding RNA (lncRNA) with a length of more than 200 nucleotides (nt) is a significant component of the ncRNA. lncRNA was initially thought to be “transcriptional noise” [5], but subsequent research has shown that lncRNA is often engaged in transcriptional, posttranscriptional, and epigenetic regulation [6–8]. In contrast to the complicated roles of the mRNA and protein, the stable character of lncRNAs represents that they may be more easily accessible as a prognostic indication [9]. Feng et al. found that iron metabolism-associated lncRNAs can be used for postoperative assessment of ovarian cancer (OC) patients and can effectively stratify risk [10]. Zheng et al. revealed that LINC01134 can interact with miR-4784 to inhibit the production of structure-specific recognition protein 1 (SSRP1) [11], consequently boosting hepatocellular carcinoma (HCC) development. In addition to solid tumors, lncRNAs have also been implicated in the development of hematologic tumors; in acute myeloid leukemia (AML), SATB1-AS1 is involved in the process of chemotherapy tolerance. In HNSCC, HOTAIR is overexpressed and regulates PTEN methylation involved in tumor development [12]. Numerous recent reviews detailed the biological significance of lncRNAs and indicated that lncRNAs may emerge as a potential therapeutic option for HNSCC in the future [13, 14]. Recently, some lncRNAs have been reported to be differentially expressed in HNSCC. The lncRNA expression varies significantly across tumors,tissues, as well as between the clinical or pathology stage. Cao et al. identified a prognostic lncRNA signature by the least absolute shrinkage and selection operator (LASSO) regression analysis [15], however, they did not validate the abovementioned signature in the external dataset. Therefore, lncRNA is a potential marker for diagnosis and prognosis compared to other clinicopathological features.

Machine learning is a branch of artificial intelligence that is used to do classification, regression, clustering, and canonical modeling [16]. Machine learning is a method for choosing the best model from a collection of alternatives that best fits a set of observations [17]. Relying on this approach, Feng et al. performed machine learning model building, such as Random forest (RF), to identify the hypoxic landscape within ovarian cancer tissues in conjunction with radiogenomics data [18]. Although a large number of previous studies have reported the association of lncRNAs with the pathogenesis and prognosis of HNSCC, no unique hub biomarkers have been identified and compounded using machine learning based on public datasets.

In this study, we hypothesized that there are lncRNA expression patterns that are strongly linked with HNSCC prognosis, which are attractive for therapeutic application. A comprehensive analysis of lncRNA expression profiles of the TCGA-HNSCC cohort with clinical information was performed to investigate the prognostic value of each lncRNA. Finally, we identified the hub marker, LINC01615, based on overall survival (OS), progress-free interval (PFI), and disease-specific survival (DSS) data, using LASSO and RF methods. The prognostic value, clinicopathological relevance, potential biological function, somatic mutations, and immune infiltration of LINC01615 at the pan-cancer level, especially in HNSCC, were explored in depth. Functionally, we validated that LINC01615 plays a pivotal role in head and neck squamous cell carcinoma cell proliferation, invasion, and migration.

## 2. Materials and Methods

### 2.1. Datasets and Preprocessing

We downloaded RNA-seq data consisting of 502 HNSCC tissues and 44 normal tissues from the TCGA database [19] (April 22, 2022; https://cancergenome.nih.gov/), normalized using the log(*x* + 1) method and converted it to the TPM format. The latest version of lncRNA genomic sequence information (April 22, 2022) was downloaded from the GENECODE database [20] to annotate lncRNA expression profiles in the TCGA-HNSCC cohort. The TCGA database was used to acquire clinical and survival data on HNSCC patients. Clinical information such as survival status (OS, PFI, and DSS), age, gender, pathological stage, and tumor grade was combined with RNA-seq ID. The expression and clinical data for LINC01615 (ENSG00000223485) were retrieved from the UCSC Xena database [21] (https://xenabrowser.net/datapages/) utilizing TCGA pan-cancer data. Pan-cancer abbreviations are included in supplementary Table S1. According to the publishing standards of TCGA, datasets are freely used for publication.

### 2.2. LASSO and Random Forest Model for Screening Biomarkers

Random forest (RF) created a huge number of decision trees using random subsamples of the training set and randomly altering the decision trees' attributes [22]. The RF model enables nonlinear effects to be modeled. Because the RF model is composed of several decision tree models, it is difficult to comprehend. The RF method is an appropriate integrated learning algorithm and machine learning technique because it has changing independent circumstances and a greater level of accuracy and sensitivity [23]. Another relevant prediction model for our research data is LASSO regression. By designing a penalty function, it is possible to compress the coefficients of the variables and so resolve the issue of overfitting the regression model. The minimal absolute shrinkage and selection operator is a regression method that is used to select and regularize variables in order to increase the predicted accuracy and interpretability of the statistical models it generates [24]. In our study, we used Cox regression to screen out prognostic biomarkers, the threshold as HR >1 and *p* value <0.05. Subsequently, the three surviving outcome-related lncRNAs overlapped to get the common lncRNAs.

### 2.3. Enrichment Analysis

The TCGA-HNSCC cohort was divided into high and low expression groups based on the median value of the LINC01615 expression, and differentially expressed genes (DEGs) were computed using p values less than 0.05 and logFC values larger than 2 as the criteria in the “limma” package. Subsequently, we conducted Gene Ontology (GO) and Kyoto Encyclopedia of Genes and Genomes (KEGG) analyses [25] using the the “clusterProfiler” package [26] (*p* value = 0.05, *q* value = 0.05). Gene set enrichment analysis (GSEA) was also carried out using the “clusterProfiler” package (nPerm = 1000, minGSSize = 10, maxGSSize = 1000, and *p* value = 0.05).

### 2.4. Immune Cell Infiltration

The ssGSEA and ESTIMATE algorithms were used to estimate immune cell infiltration scores in the TCGA-HNSCC cohort [27]. To examine the amount of immune cell infiltration, the whole sample was separated into two groups based on the median LINC01615 expression, and the correlation between LINC01615 and different immunological scores was determined using spearman analysis.

### 2.5. Nomogram and Predictive Performance Evaluation

Cox regression analysis, both univariate and multivariate, was conducted to determine if LINC01615 might be an independent prognostic factor. Additionally, nomogram [28] and calibration plots for the predicting 1-year, 3-year, and 5-year OS were constructed using the “rms” package. The calibration plots analyzed visually by mapping the predicted probability from the nomogram to the actual rates. The bootstrap approach was used to calculate 1000 resamples. Additionally, receiver operating characteristic (ROC) curves were utilized to determine the predictive accuracy in survival and diagnosis prediction.

### 2.6. Tumor Mutational Burden (TMB) in Different Groups

Somatic mutations from TCGA-HNSCC were retrieved from the TCGA database. The tumor mutation burden (TMB) was estimated using TCGA somatic mutation data for each tumor as the number of mutated bases per million bases. The whole sample was separated into two groups based on the median LINC01615 expression, and the “maftools” package [29] was used to explore the difference of the mutation frequency in the high and low group.

### 2.7. Comprehensive Index in Pan-Cancer

To better understand the genomic heterogeneity caused by different LINC01615 expression levels, we explored the Spearman correlation coefficient between the LINC01615 expression and several indicators in pan-cancer, including microsatellite instability (MSI), homologous recombination deficiency (HRD), and TMB. Based on previous studies [30, 31], we downloaded data for genomic heterogeneity.

### 2.8. Cell Lines and Culture

Two laryngeal squamous cell carcinoma cell lines, HEP-2 and TU212, were obtained from the National Collection of Authenticated Cell Culture of Chinese Academy of Sciences. All cells were cultured with DMEM containing 10% fetal bovine serum (FBS; Gibco, Gaithersburg, MD, USA), 100 U/ml penicillin, and 100 *μ*g/ml streptomycin in an incubator with 95% humidified air containing 5% CO_2_ at 37°C.

### 2.9. siRNA Transfection

Cells (1 × 106 per well in a 6-wells plate) were transfected with 50 nM of the following siRNAs: control siRNA (5′-UAAGGCUAUGAAGAGAUACUU-3′), LINC01615 siRNA1 (5′-CUAAUCCCCACGUUGACUGCUU-3′), and LINC01615 siRNA2 (5′-CUGGCAACGCCUGCUCUCUGCUU-3′) for 72 h according to the manufacturer's instructions. Four-eight hours later, cells were trypsinized and seeded for the different functional assays.

### 2.10. MTT Assay

After siRNA transfection for 48 h, 2 × 10^4^ cells were seeded into a 96-wells plate and incubated with 10 mL MTT of 0.5 mg/ml at 37 *μ*C for 4 h on days 0, 1, 2, 3, and 4. The absorbance was measured using a spectrophotometer (Tecan Group Ltd., Switzerland) at 570 nm.

### 2.11. Transwell Invasion and Migration Assay

Twenty-four-well plate inserts (pore size: 8 *μ*m) were precoated with 25 *μ*l of Matrigel matrix (diluted at 1 : 3 with basic culture medium) and incubated at the cell culture incubator to form a gel. Then, 5 × 10^4^ cells were suspended with 250 *μ*L and added into the upper transwell chamber. The lower chamber was filled with a 500 *μ*L culture medium with 10% FBS. After incubation at 37°C for 24 h, noninvaded cells on the upper chamber were scraped with a cotton swab. Invaded cells were fixed with 100% methanol and stained with 0.05% crystal violet. Images were taken, and the invaded cells were counted manually. The experiments were performed independently in triplicate for each cell line. For the migration assay, the inserts were directly suspended with 2 × 10^4^ cells without the Matrigel matrix.

### 2.12. Statistical Analysis

All statistical analyses were performed using R software (v.4.1.1). Detailed statistical methods are covered in the above section. *p* <0.05 was considered statistically significant.

## 3. Results

### 3.1. Screening of LINC01615 Using LASSO and Random Forest

**Figure 1:**
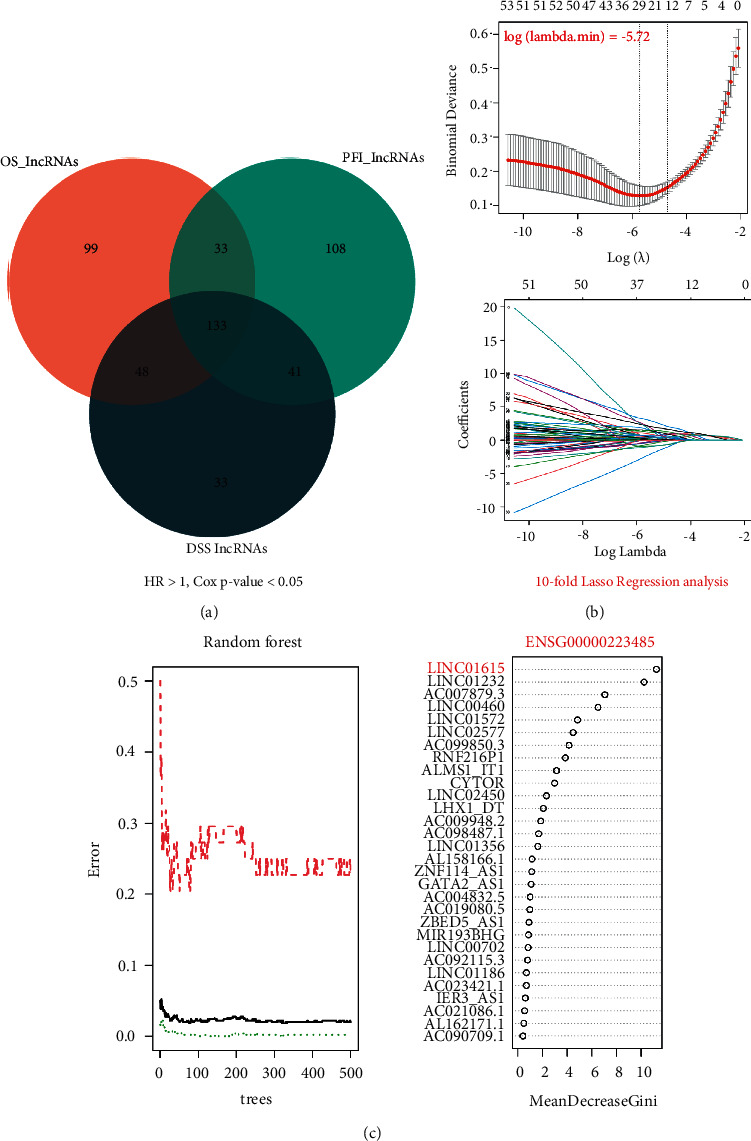
Screening of the hub lncRNA using machine learning. (a) The Venn plot of the heatmap of three survival outcomes (overall survival, progress-free interval, and disease-specific survival)-related lncRNAs. (b) LASSO regression analysis in 133 common-lncRNAs. (c) The RF model was constructed by 33 lncRNAs from the LASSO model to calculate the gene importance score.

Firstly, we used Cox regression analysis to screen prognostic lncRNAs using different dependent variables (OS, PFI, and DSS). We overlapped the abovementioned lncRNAs to get the 133 common lncRNAs with multiple prognostic significance (Figure 1(a)). To further eliminate prognostic lncRNAs, LASSO regression (10-fold) was used to further screen out 33 lncRNAs, and −5.72 as log (minimum lambda) value (Figure 1(b)). The RF model (*n*tree = 500) constructed by 33 lncRNAs from the LASSO model was used to calculate the gene importance score (Figure.  1(c)). Finally, the combined machine learning algorithm screened out the hub lncRNA with the highest importance: LINC01615.

### 3.2. Expression and Prognostic Analysis of LINC01615 in the Pan-Cancer Level

**Figure 2:**
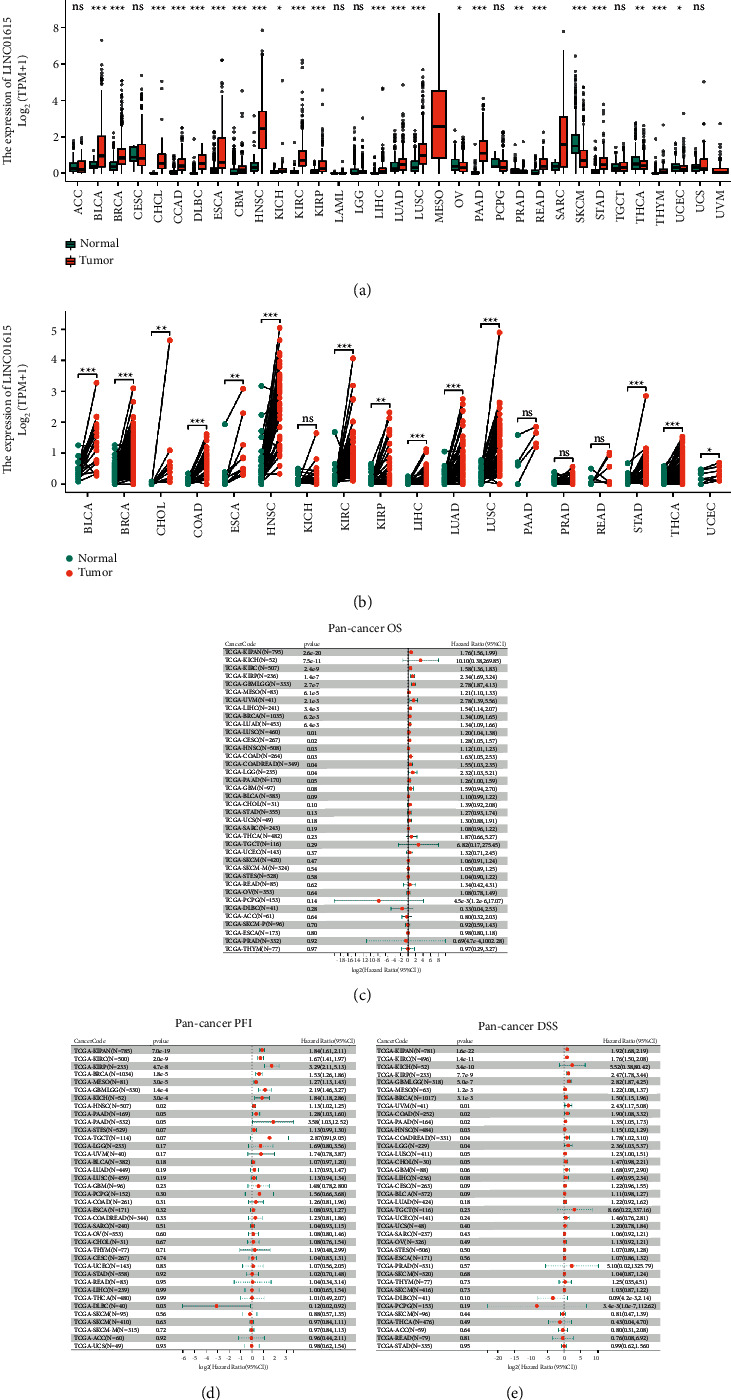
Differential expression analysis and prognostic analysis in pan-cancer. (a) Differential expression analysis of nonpaired samples. (b) Differential expression analysis of paired samples. (c) The forest plot of OS in pan-cancer analysis. (d) The forest plot of PFI in pan-cancer analysis. (e) The forest plot of DSS in pan-cancer analysis.

We used LINC01615 (ENSG00000223485) retrieved from the UCSC Xena database for conducting different expression analyses of nonpaired or paired samples. Based on our results, we revealed that LINC01615 was overexpressed in most tumors compared to normal tissues, while it was less expressed in OV, PRAD, SKCM, THCA, and UCEC (Figure 2(a)). However, in the paired samples, LINC01615 was highly expressed in the vast majority of cancers, and only KICH, PRAD, PEAD, and PAAD were not significant (Figure 2(b)). Importantly, LINC01615 was significantly upregulated in HNSCC compared with normal tissues. The differences in the LINC01615 expression levels in different tumor types may reflect different underlying functions and mechanisms. In pan-cancer survival analysis, LINC01615 predicted poor tumor prognosis. In the forest plot of OS (Figure 2(c)), LINC01615 was overwhelmingly present as a risk factor, while in the PFI analysis (Figure 2(d)), it was only present as a protective factor in DLB. Similarly, in DSS analysis, LINC01615 could also present as a risk factor in different cancers (Figure 2(e)).

### 3.3. Clinical Application Analysis in HNSCC Patients

**Figure 3:**
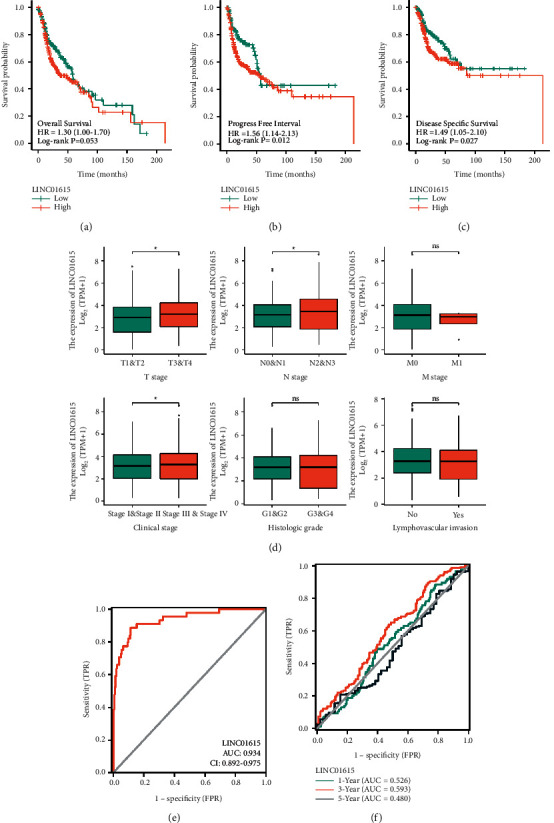
Clinical correlation analysis in HNSCC. (a) Kaplan–Meier survival analysis of different LINC01615 expression groups in overall survival. (b) Kaplan–Meier survival analysis of different LINC01615 expression groups in the progress-free interval. (c) Kaplan–Meier survival analysis of different LINC01615 expression groups in disease-specific survival. (d) Expression levels of LINC01615 in different clinical features, including T stage, N stage, M stage, clinical stage, histologic grade, and lymphovascular invasion. (e) ROC curve for diagnosis. (f) ROC curve for survival prediction in 1-year, 3-years, and 5-years. ^*∗*^*p* < 0.05, ^*∗∗*^*p* < 0.01.

**Figure 4:**
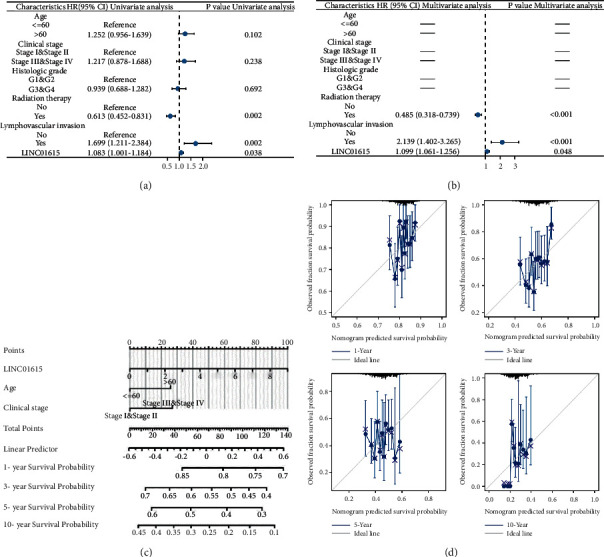
Construction of nomogram. (a) The forest plot of univariate Cox regression analysis. (b) The forest plot of multifactorial Cox regression analysis. The horizontal lines mean that the value cannot be calculated. (c) Nomogram. (d) Calibration curve for 1-year, 3-years, 5-years, and 10-years.

Kaplan–Meier analysis revealed that OS (Figure 3(a)), PFI (Figure 3(b)), and DSS (Figure 3(c)) were all shorter in the high-LINC01615-expressing group compared to the low-LINC01615-expressing group, while the probability of mortality and disease progression was increased. Meanwhile, clinical correlation analysis revealed that the expression of LINC01615 increased with increasing the T stage, N stage, pathology grade, and clinical stage (Figure 3(d)). Only eight patients in the TCGA-HNSCC cohort were at the M1 stage, which may explain why no difference was seen. Additionally, the receiver operating characteristic curve demonstrated that LINC01615 had a perfect predictive value for tumor diagnosis (Figure 3(e)). However, when paired with the time variable, the AUC value of survival was low; 1-year, 3-year, and 5-years were 0.526, 0.593, and 0.480, respectively (Figure 3(f)). Next, the Cox regression analysis confirmed that LINC01615 was an independent risk factor (Figures 4(a) and 4(b)). We constructed a nomogram by combining the LINC01615 expression with classical clinical indicators (Figure 4(c)). The calibration curve showed good predictive performance in 1 year, 3 years, 5 years, and 10 years (Figure 4(d)). Hence, we discovered that when clinical indicators were combined with LINC01615, the visualization model was more applicable to clinical practice.

### 3.4. Enrichment Analysis and Immunological Characteristics Based on the LINC01615 Expression

**Figure 5:**
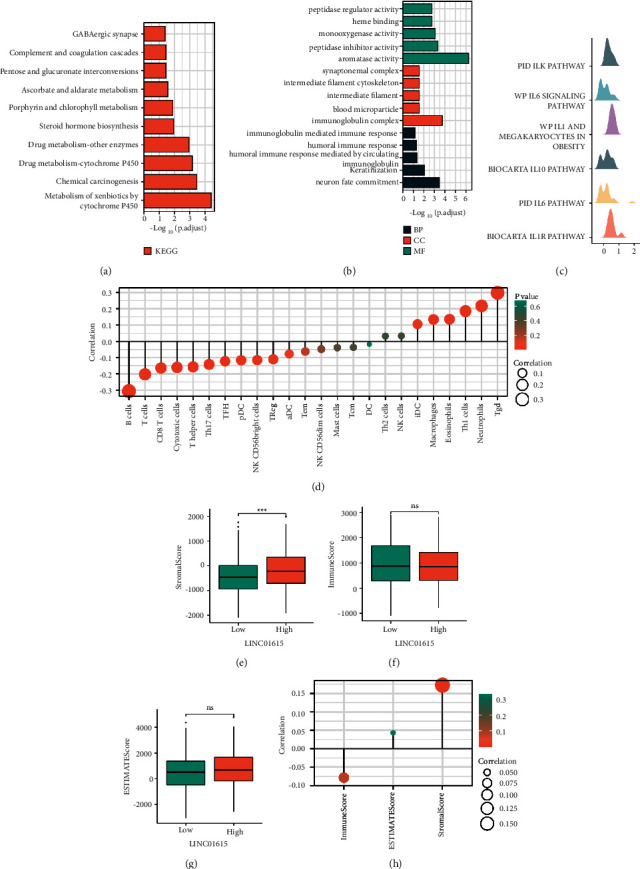
Enrichment analysis. (a) Top 10 terms of KEGG analysis. (b) Top 5 terms in each section of GO analysis. (c) The results of GSEA. (d) Correlation analysis between the LINC01615 expression and results of the ssGSEA algorithm. (e) Difference of the stromal score in different LINC01615 groups. (f) Difference of the immune score in different LINC01615 groups. (g) Difference of the ESTIMATE score in different LINC01615 groups. (h) Correlation analysis between the LINC01615 expression and results of the ESTIMATE algorithm.

To elucidate the reasons for the altered biological functions caused by the differential LINC01615 expression, we performed enrichment analyses. The whole cohort was divided into high- and low-expression- groups based on the median value of the LINC01615 expression, and differentially expressed genes (DEGs) were identified in the “limma” package. Finally, we screened out 209 DEGs for GO and KEGG analyses. Among them, KEGG analysis showed that the metabolism or immune-related pathways were enriched, such as GABAergic synapse, complement and coagulation cascades, and cytochrome P450 metabolism (Figure 5(a)). As evidenced by the significant enrichment of the immunoglobin complex, , the humoral immune response mediated by the circulating immunoglobin also confirmed the abovementioned possibility in GO analysis (Figure 5(b)). GSEA analysis showed that LINC01615 was associated with most of the immune pathways, such as the IL6 signaling pathway, IL1 and megakaryocytes in obesity, and the biocarta IL10 pathway. (Figure 5(c)). The LINC01615 overexpression was shown to be related with increased stromal scores in the ESTIMATE algorithm (Figure 5(h)), while being negatively associated with the majority of natural killer cells, such as CD8 T cells(Figure 5(d)). The high expression of LINC01615 may represent a higher stromal score (Figure 5(e)), but it was not different in the immune score (Figure 5(f)) and the ESTIMATE score (Figure 5(g)). These results indicated that LINC01615 may be involved in the regulation of lymphocyte recruitment and immunological infiltration in HNSCC. Therefore, we have performed an in-depth analysis of the possible immune regulation involved in LINC01615 (Figures S1-S2), including chemokine, receptor, MHC, immune-inhibitor, immune-stimulator, and immune checkpoints. The results of the pan-cancer analysis showed that the expression of most immunomodulatory factors and immune checkpoint mRNA was positively correlated with LINC01615; however, the LINC01615 expression in HNSCC was negatively correlated with immune-stimulator genes, such as TMIGD2, CD40LG, and TNFRSF13B,.

### 3.5. Genomic Heterogeneity Based on the LINC01615 Expression in the Pan-Cancer Level

**Figure 6:**
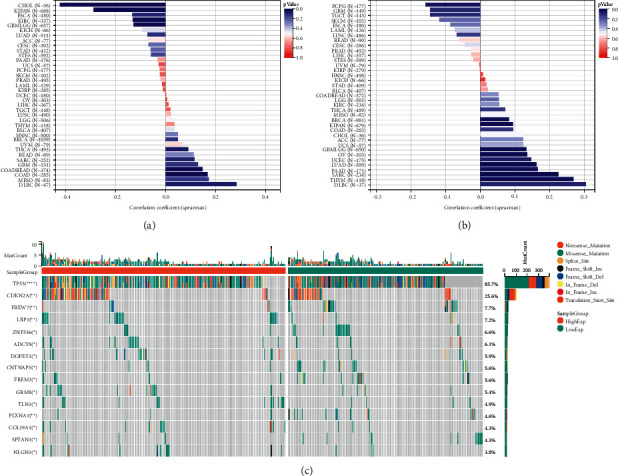
Genomic heterogeneity in pan-cancer analysis. (a) Correlation analysis between the LINC01615 expression and the MSI score. (b) Correlation analysis between the LINC01615 expression and results of the TMB score. (c) Differences of somatic mutations in different LINC01615 expression levels.

Considering the LINC01615 expression results in the activation of different pathways, we explored the genomic heterogeneity (MSI, HRD, and TMB) based on the LINC01615 expression in the pan-cancer level. Firstly, we explored the correlation analysis between the LIN01615 expression and the MSI score (Figure 6(a)). DLBC had the strongest positive correlation with the MSI score, and CHOL had the strongest negative correlation with the MSI score; however, the correlation between the LINC01615 expression and the MSI score was not statistically significant in HNSCC. In the analysis of the TMB score, similarly, the correlation between the LINC01615 expression and the MSI score was not statistically significant in HNSCC (Figure 6(b)). In HRD score analysis, there was a strong positive correlation between the LINC01615 expression and score (Figure S3). Finally, we found that patients in the high expression group had a higher frequency of mutations in TP53 and CDKN2A, compared to the low expression group (Figure 6(c)).

### 3.6. Silencing of LINC01615 Suppresses Cell Proliferation, Invasion, and Migration in HEP-2 and TU212 Cells

**Figure 7:**
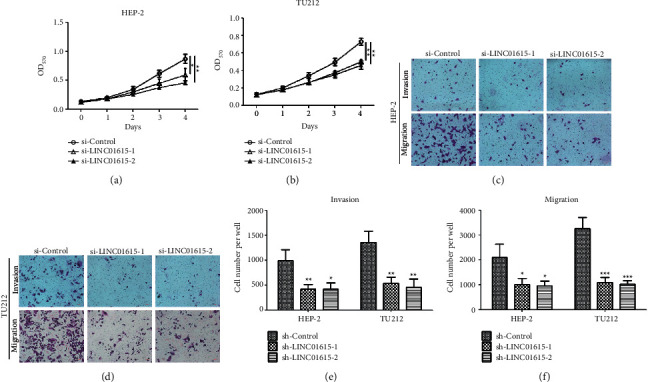
LINC01615 knockdown inhibits cell proliferation, invasion, and migration in HEP-2 and TU212 cells. (a, b) Cell proliferation was detected in the HEP-2 (a) and TU212 (b) cells after miR-control siRNA or LINC01614 siRNA transfection by the MTT assay. (c, d) The effect of LINC01615 silencing on cell invasion and migration are examined by the transwell assay. (e, f) Count of invaded and migrated cell numbers of 6 visual fields. ^*∗*^*p* < 0.05, ^*∗∗*^*p* < 0.01, ^*∗∗∗*^*P* < 0.001, vs. control siRNA.

To validate the function of LINC01615 on the biological behavior of head and neck squamous cell carcinoma, we transfected two independent siRNAs targeting LINC01615 in laryngeal squamous cell carcinoma cells HEP-2 and TU212. The MTT assay demonstrated that the numbers of cells in the LINC01615 siRNA1 and siRNA2 groups was significantly decreased compared with those in the control siRNA group (Figures 7(a) and 7(b)). The effect of LINC01615 silencing on the invasion and migration of HEP-2 and TU212 cells was assessed using transwell migration and Matrigel invasion assays. We found that silence of LINC01615 obviously decreased the invasion and migration abilities of these cells (Figures 7(c)–7(f)).

## 4. Discussion

HNSCC is one of the most common cancers, and despite significant breakthroughs in clinical research and novel medicines, the overall survival rate remains poor [32]. lncRNAs have garnered considerable interest in recent years as new regulatory elements with a variety of biological roles [33]. Numerous lncRNAs have been implicated in the etiology and prognosis of HNSCC, however, comprehensive prognostic analysis of lncRNAs at the omics analysis of HNSCC is absent. As a consequence, we performed Cox regression analysis to screen potential prognostic genes utilizing various survival outcomes as dependent variables, resulting in the identification of 133 lncRNAs with multiple prognostic significance. Subsequently, the machine learning algorithm was used to further screen the hub gene with the highest importance in the random forest model: LINC01615. Clinical correlation analysis revealed that the expression of LINC01615 increased with increasing the T stage, N stage, pathology grade, and clinical stage. Only eight patients in the TCGA-HNSCC cohort were at the M1 stage, which may explain why no difference was seen. Meanwhile, the Kaplan–Meier analysis revealed that OS, PFI, and DSS were all shorter in the high LINC01615-expressing group compared to the low LINC01615-expressing group, while the probability of mortality and disease progression was increased. Additionally, the receiver operating characteristic curve demonstrated that LINC01615 had a perfect predictive value for tumor diagnosis. However, when paired with the time variable, the AUC value of survival was low; 1-year, 3-year, and 5-years were 0.526, 0.593, and 0.480, respectively. Hence, we incorporated other clinical indicators to construct the nomogram. We discovered that when clinical indicators were combined, the visualization model is more applicable to clinical practice. Based on the abovementioned findings, we believe that the expression of LINC01615 could be used as a predictor of HNSCC prognosis.

LncRNAs have received increasing attention for their function in regulating innate and adaptive immune cell responses [34, 35]. Therefore, we have performed an in-depth analysis of the possible immune regulation involved in LINC01615, including chemokine, receptor, MHC, immune-inhibitor, immune-stimulator, and immune checkpoints. The results of the pan-cancer analysis showed that the expression of most immunomodulatory factors and the immune checkpoint mRNA were positively correlated with LINC01615; however, the LINC01615 expression in HNSCC was negatively correlated with immune-stimulator genes, such as TMIGD2, CD40LG, and TNFRSF13B. Immune cell infiltration is a hallmark of the host to tumor cells and is strongly connected with the genesis and progression of cancer. Our findings revealed that the expression of LINC01615 was strongly related with immune cell infiltration in HNSCC. A low proportion of M0 macrophages and a high level of Treg expression have been associated with a favorable OS and DFI in HNSCC patients [36]. The LINC01615 overexpression was shown to be related with increased stromal scores in the ESTIMATE algorithm, while being negatively associated with the majority of natural killer cells, such as CD8 T cells. These results indicated that LINC01615 may be involved in the regulation of lymphocyte recruitment and immunological infiltration in HNSCC.

To elucidate the reasons for the altered biological functions caused by the differential LINC01615 expression, we performed enrichment analyses. Among them, GSEA analysis showed that LINC01615 is associated with most of the immune pathways, such as the IL6 signaling pathway, IL1 and megakaryocytes in obesity, and the biocarta IL10 pathway. As evidenced by the significant enrichment of immunoglobin complex, humoral immune response mediated by the circulating immunoglobin also confirmed the abovementioned possibility in GO analysis. Unfortunately, there are very few basic studies on LINC01615, and only one study has identified mRNA-lncRNA pairs by gene co-expression analysis elucidating that LINC01615 may have significant implications for HNSCC patients [37]. Interestingly, LINC01615 may be a lncRNA associated with ferroptosis [38, 39], and in gastric cancer cell lines, most LINC01615 is enriched in the cytoplasm. In addition, only three studies have investigated the regulatory role of LINC01615 in depth. LINC01615 functions as an oncogene and is involved in cell proliferation, apoptosis, invasion, and migration in colorectal cancer cells [40]. LINC01615 competitively binds with miR-3653-3p to regulate ZEB2 and promote the carcinogenesis of colon cancer cells [41]. Particularly, Dong et al. revealed that LINC01615 potentially affected the extracellular matrix and had further impacts on the metastasis of hepatocellular carcinoma [42], the findings were consistent with our research. The only method we used was bioinformatics and machine learning to predict the potential indicative value in HNSCC, so further experiments are needed in the future.

In a pan-cancer analysis of genomic heterogeneity, we explored the expression level and the prognostic value of LINC01615 using TCGA data from the UCSC Xena database. Based on our results, we revealed that LINC01615 was overexpressed in most tumors compared to normal tissues, while it was less expressed in OV, PRAD, SKCM, THCA, and UCEC. The differences in LINC01615 expression levels in different tumor types may reflect different underlying functions and mechanisms. In pan-cancer survival analysis, LINC01615 predicted poor tumor prognosis. In the forest plot of OS, LINC01615 was overwhelmingly present as a risk factor, while in the PFI analysis, it was only present as a protective factor in DLB. These results suggested that LINC01615 may be a prognostic biomarker to predict the prognosis for most tumor patients. More importantly, the expression status of LINC01615 and MSI, HRD, and TMB in different tumors also showed significant heterogeneity. Functionally, we furtherly validated that LINC01615 plays a pivotal role in head and neck squamous cell carcinoma cell proliferation, invasion, and migration. There are major limitations, the first being the identification and validation in the TCGA dataset only in our study. In addition, LINC01615 needs further confirmation of function in future basic studies. In conclusion, LINC01615 may play an important role in the prostromal cell enrichment and immunosuppressive state and serve as a valuable prognostic biomarker in HNSCC.

## Data Availability

The following information was supplied regarding data availability: Data are available at the TCGA database (https://portal.gdc.cancer.gov/).

## Supplementary Materials

Supplementary Materials Supplementary Figure 1: Spearman correlation analysis between LINC01615 and immune indicators. Supplementary Figure 2:Spearman correlation analysis between LINC01615 and the immune checkpoint. Supplementary Figure 3: Spearman correlation analysis between LINC01615 and the HRD score. Supplementary Table 1: full names of abbreviations in pan-cancer.
