# Supplementary material for: Comprehensive Analysis of LINC01615 in Head and Neck Squamous Cell Carcinoma: A Hub Biomarker Identified by Machine Learning and Experimental Validation
Source: J Oncol. 2022 Jun 27;2022:5039962. doi: 10.1155/2022/5039962 (PMC9252709; doi:10.1155/2022/5039962)
Supplement: Supplementary Materials — Supplementary Figure 1: Spearman correlation analysis between LINC01615 and immune indicators. Supplementary Figure 2:Spearman correlation analysis between LINC01615 and the immune checkpoint. Supplementary Figure 3: Spearman correlation analysis between LINC01615 and the HRD score. Supplementary Table 1: full names of abbreviations in pan-cancer. [file 5039962.f1.pdf]

Supplemental figure 1

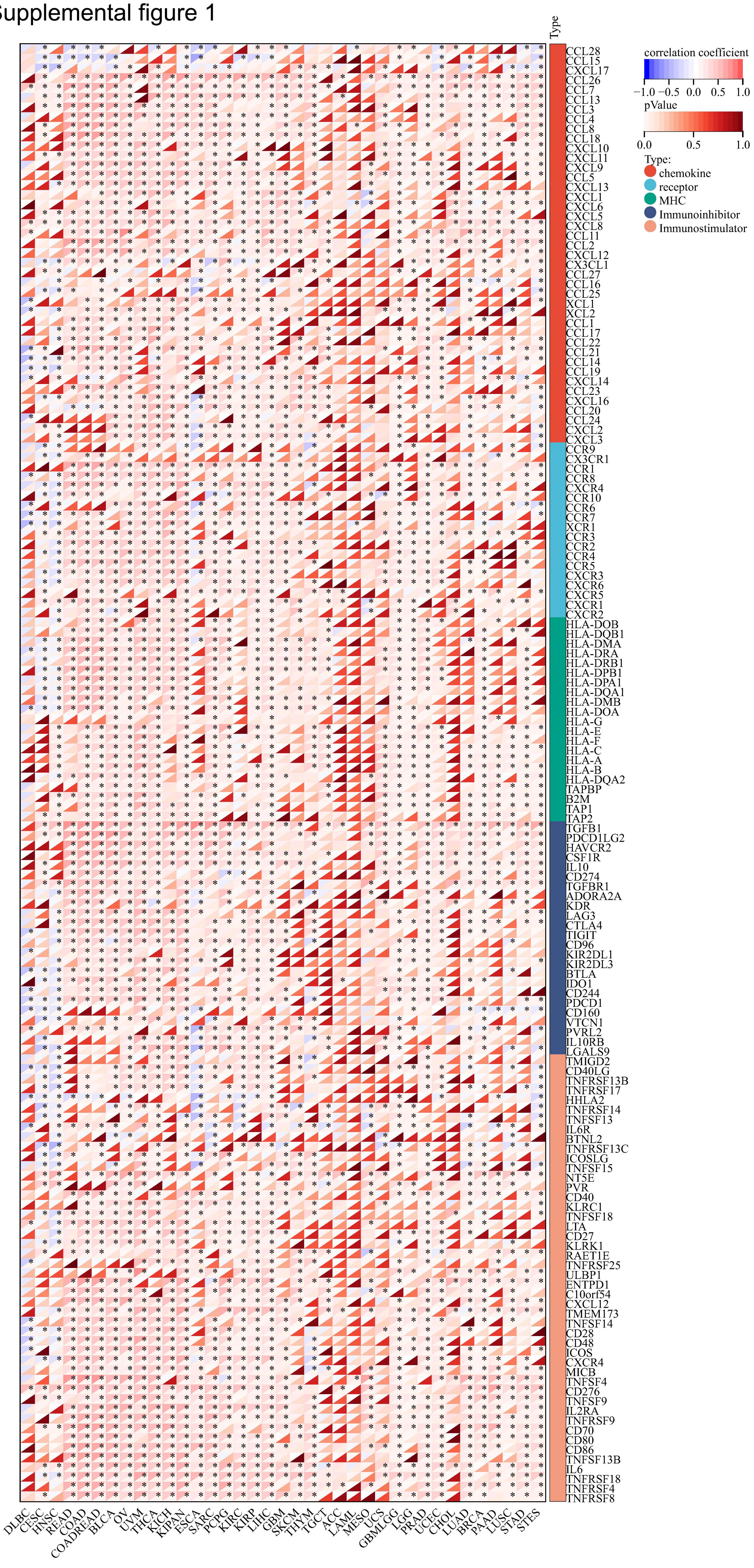

Supplemental figure 2

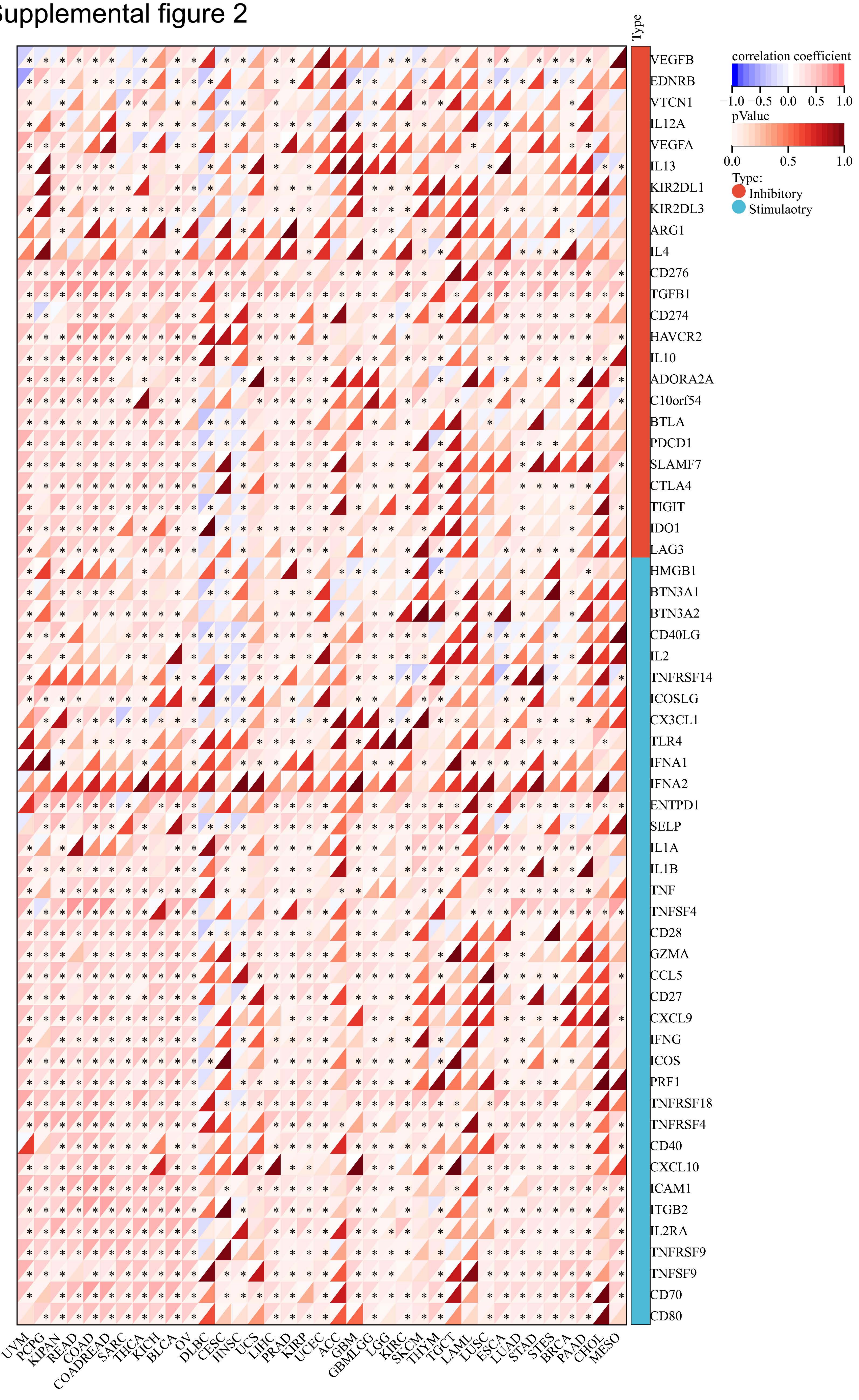

Supplemental figure 3

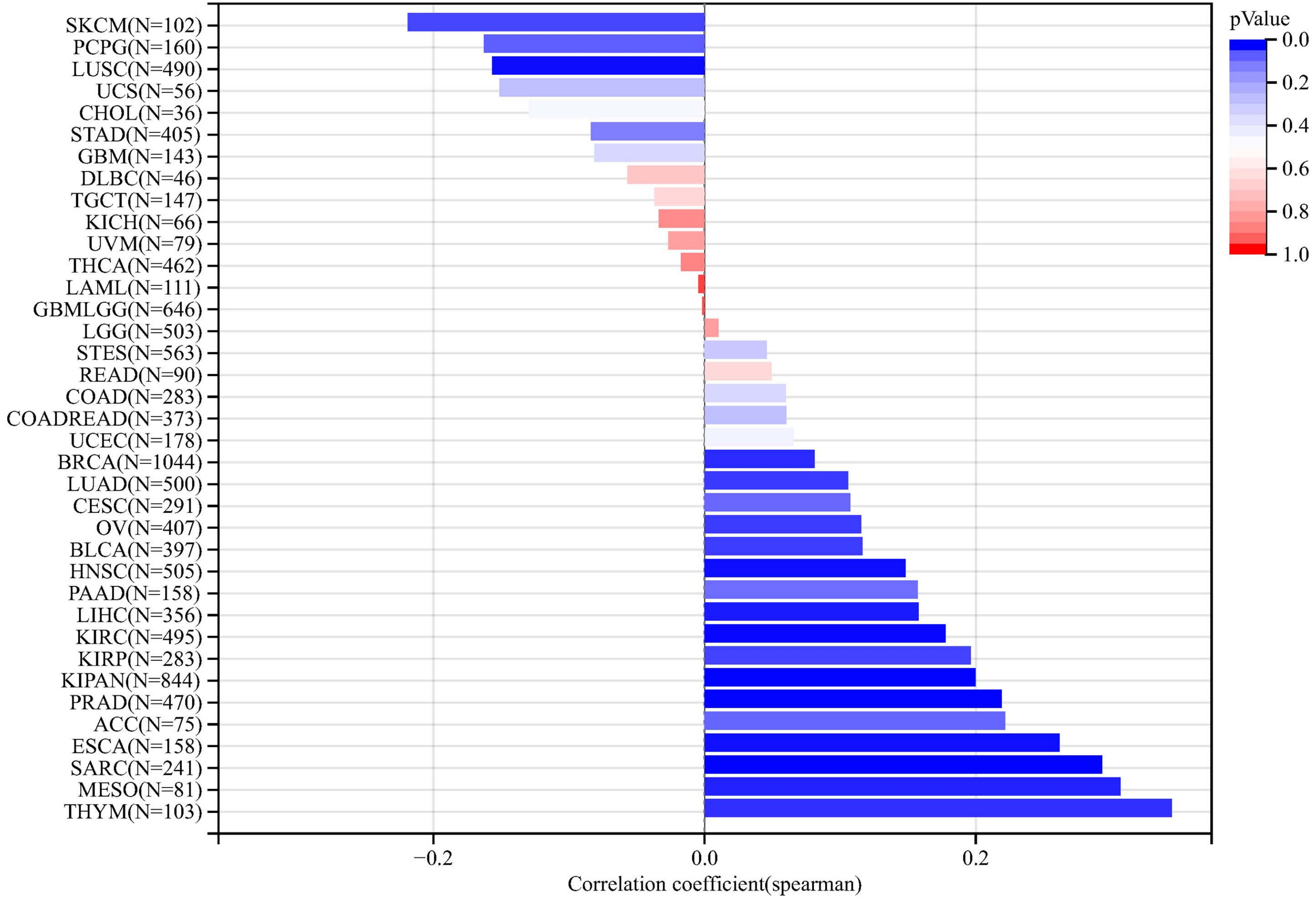

Supplementary Table 1: Full names of abbreviations

ACC	Adrenocortical carcinoma
BLCA	Bladder Urothelial Carcinoma
BRCA	Breast invasive carcinoma
CESC	Cervical squamous cell carcinoma and endocervical adenocarcinoma
CHOL	Cholangiocarcinoma
COAD	Colon adenocarcinoma
DLBC	Lymphoid Neoplasm Diffuse Large B-cell Lymphoma
ESCA	Esophageal carcinoma
GBM	Glioblastoma multiforme
HNSC	Head and Neck squamous cell carcinoma
KICH	Kidney Chromophobe
KIRC	Kidney renal clear cell carcinoma
KIRP	Kidney renal papillary cell carcinoma
LAML	Acute Myeloid Leukemia
LGG	Lower Grade Glioma
LIHC	Liver hepatocellular carcinoma
LUAD	Lung adenocarcinoma
LUSC	Lung squamous cell carcinoma
MESO	Mesothelioma
OV	Ovarian serous cystadenocarcinoma
PAAD	Ovarian serous cystadenocarcinoma
PCPG	Pheochromocytoma and Paraganglioma
PRAD	Prostate adenocarcinoma
READ	Rectum adenocarcinoma
SARC	Sarcoma
SKCM	Skin Cutaneous Melanoma
STAD	Stomach adenocarcinoma
TGCT	Testicular Germ Cell Tumor
THCA	Thyroid carcinoma
THYM	Thymoma
UCEC	Uterine Corpus Endometrial Carcinoma
UCS	Uterine Carcinosarcoma
UVM	Uveal Melanoma
